# Monomerization of ALK Fusion Proteins as a Therapeutic Strategy in *ALK*-Rearranged Non-small Cell Lung Cancers

**DOI:** 10.3389/fonc.2020.00419

**Published:** 2020-04-02

**Authors:** Noriko Hirai, Takaaki Sasaki, Shunsuke Okumura, Yoshinori Minami, Shinichi Chiba, Yoshinobu Ohsaki

**Affiliations:** ^1^Respiratory Center, Asahikawa Medical University Hospital, Asahikawa, Japan; ^2^Center for Advanced Research and Education, Asahikawa Medical University, Asahikawa, Japan

**Keywords:** coiled-coil domain, EML4-ALK rearrangement, lung cancer, oncogene fusion proteins, peptide synthesis

## Abstract

**Objective:** Oncogenic echinoderm microtubule-associated protein-like 4 *(EML4)-*anaplastic lymphoma kinase (*ALK*) (EML4-ALK) fusion proteins found in non-small cell lung cancers (NSCLC) are constitutively phosphorylated and activated by dimerization via the coiled-coil domain (cc) within EML4. Here, we investigated whether disruption of ALK fusion protein oligomerization via competitive cc mimetic compounds could be a therapeutic strategy for EML4-ALK NSCLC.

**Methods:** A Ba/F3 cell model was created that expressed an ALK intracellular domain in which the dimer/monomer state is ligand-regulated. This novel cell model was used to investigate the effect of disrupting ALK fusion protein oligomerization on tumor cell growth *in vitro* and *in vivo* using nude mice. Subsequently, the antiproliferative effects of endogenous cc domain co-expression and mimetic cc peptides were assayed in EML4-ALK cancer cell lines.

**Results:** Cells induced to express monomeric ALK *in vitro* did not survive. When transplanted into mice, induction of monomers abrogated tumor formation. Using a fluorescent protein system to quantify protein-protein interactions of EML4-ALK and EML4cc, we demonstrated that co-expression of EML4cc suppressed EML4-ALK assembly concomitant with decreasing the rate of tumor growth *in vitro* and *in vivo*. In EML4-ALK cancer cell lines, administration of synthetic EML4cc peptide elicited a decrease of phosphorylation of ALK leading to reduction in tumor cell growth.

**Conclusions:** Our findings support the monomerization of ALK fusion proteins using EML4cc peptides for competitive inhibition of dimerization as a promising therapeutic strategy for EML4-ALK NSCLC. Further studies are warranted to explore the use of specific cc peptide as a therapeutic option for other lung cancers harboring driver fusion genes containing a cc or oligomerization domain within the fusion partner.

## Introduction

Several genetic alterations, including mutation or rearrangements, were recently identified as molecular targets of therapies for lung cancers with promising results (1–3). The echinoderm microtubule-associated protein-like 4 (*EML4*)-anaplastic lymphoma kinase (*ALK*) fusion oncogene, arising from an inversion on chromosome 2, was discovered in 2007 and is found in 5% of non-small cell lung cancers (NSCLC) worldwide (4–6). ALK tyrosine kinase inhibitors (ALK-TKI) have anti-tumor activities in NSCLC with ALK rearrangements, but complete cancer control has not been achieved due to acquired resistance (1, 3).

Gain-of-function mutations (e.g., F1174L or R1275Q) in ALK were first identified in neuroblastoma and induce constitutive autophosphorylation of ALK (7–9). Some of these activating mutations also confer secondary resistance against ALK-TKI in ALK-rearranged cancers (3, 6). In contrast, the EML4-ALK fusion which is the most frequent ALK rearrangement in NSCLC, requires homodimerization via a trimeric coiled-coil (cc) domain at the amino-terminal end of EML4 for constitutive ALK activation (4, 5, 10–12). All reported EML4-ALK variants, including even the shortest variant 5a/b, contain the EML4-cc domain; in the non-EML4 ALK fusion partners reported in NSCLC (e.g., KIF5B-, TPM3-, TPM4-, or TPR-) the amino-terminal fragment of the fusion partner necessarily contains cc domains or oligomerization domains (13–15).

Previous studies of oligomerization domain-targeting therapies focused on leukemia caused by the Bcr-Abl (Philadelphia chromosome) t(9;22) chromosome translocation (16). Formation of Bcr-Abl tetramers through the amino terminal oligomerization domain of Bcr promotes activation of the Abl kinase (17–20). Deletion or overexpression of the Bcr-Abl oligomerization domain, or treatment with mimicking peptides that disrupt oligomerization of Bcr-Abl, has demonstrated distinct antiproliferative activities in leukemia cell lines and in some strains resistant against Abl kinase inhibitors (18, 21–25). Blocking oligomerization of fusion proteins using the structure of cc domain itself is a potential therapeutic approach to treat cancers of ALK-rearrangements in which the ALK fusion partner has a cc domain; however, to date, this approach has not been experimentally tested. Such peptide therapies may provide a new therapeutic strategy for EML4-ALK cancers that are resistant to TKI.

In this study, we hypothesized that overexpression of the EML4-cc domain would compete or interfere with oligomerization of the full-length EML4-ALK fusion protein. Consequently, we postulated that ALK kinase activity is dependent on EML4-ALK dimerization and that cc peptides would provide appreciable antiproliferative activities in ALK-rearranged cancers. To test these hypotheses, we investigated the effects of ALK fusion protein monomerization *in vitro* and *in vivo*, and cc peptide treatment for the EML4-ALK-positive cells in cultured Ba/F3 and EML4-ALK-positive cancer cell lines.

## Materials and Methods

### Cell Line Culture and Transfection

Murine pro-B lymphocyte derived Ba/F3 cells (RCB0805, RIKEN BRC, Tsukuba, Japan), whose survival and growth depend on interleukin-3 (IL-3) supplementation, were used. Cells were subcultured twice a week in a double sealed flask with RPMI 1640 medium containing 4.5% L-glutamine (Gibco Invitrogen #11875-093, Gibco, Thermo Fisher Scientific, Waltham, MA, USA), 10% fetal bovine serum (FBS) (FB-1365/500, Biosera, Boussens, France) and 10% WEHI-3B (RCB2853, RIKEN BRC) conditioned medium to supply IL-3. Conditioned medium was prepared by centrifuging and filtering the supernatant of confluent WEHI-3B cells.

The H3122 cell strain of human derived *EML4-ALK variant 1* (consisting of *EML4 exon 1-13* fused to *ALK exon 20-29*, kindly provided by Dr. Pasi A. Jänne at Dana-Farber Cancer Institute, Boston, MA, USA) and A549 cell strain of human *KRAS* mutant NSCLC (American Type Culture Collection, Manassas, VA, USA) were trypsinized and subcultured twice a week in RPMI 1640 medium containing 4.5% L-glutamine and 10% FBS under adherent conditions. Ba/F3 cells stably expressing *EML4-ALK variant 1* (Ba/F3 EML4-ALK_wt) or *EML4-ALK variant 1* with the F1174L mutation (Ba/F3 EML4-ALK_mF1174L) were provide by Drs. Katayama and Uchibori from the Japanese Foundation For Cancer Research. These strains were subcultured in the medium described above without IL-3.

Cell growth was determined by MTS assay using CellTiter 96® AQueous One Solution Cell Proliferation Assay (G3580, Promega, Madison, WI, USA), which measures bioreduction of MTS into a soluble formazan that was quantified in a microplate reader at 490 nm. Cell titer-Glo (G7570, G7571, Promega, Madison, WI, USA) was used to evaluate the growth of H3122 cells treated with the combination of Alectinib and cc peptides.

### Transfections

The iDimerize inducible Homodimer system (#635068, TAKARA bio, Shiga, Japan) containing pHom-1 vectors and B/B homodimerizer (AP20187, hereafter called B/B) was used to induce dimerization of a protein fused to a DmrB domain. pHom-1 was linearized with EcoR1 (#1040A, TAKARA bio) and the intracellular domain of *ALK* (1687 bases from the second base of *ALK* exon 20 to the 696th base of exon 29) was ligated to the C-terminus of the *DmrB* domain using In-Fusion cloning (#639633, Clontech TAKARA bio, Shiga, Japan). This construct was transformed into *E. coli* (DH5α) and the resulting clone was introduced into Ba/F3 cells (hereafter called Ba/F3 DmrB-ALK_wt). The C-terminus of ALK was epitope-tagged with hemagglutinin (HA; 5′-TATCCGTACGACGTACCAGACTACGCA-3′) for protein purification. Ba/F3 cells stably expressing the ALK intracellular domain of the F1174L mutant (ALK c.3522C>A) fused to the *DmrB* domain (hereafter called DmrB-ALK_mF1174L) were created using site-directed mutagenesis (#200519 Agilent Technologies Inc., Santa Clara, CA, USA). Transfection was performed by electroporation using 2 μg DNA for 2 × 10^6^ cells (Nucleofector™ 2b, VCA-1003, LONZA Tokyo, Japan). After electroporation, Ba/F3 cells were immediately transferred to medium containing IL-3 and incubated for 4 h. IL-3 was removed after this incubation period and cells were cultured with 10 nM B/B for ~7 days until stably growing under continuous administration of B/B independent of IL-3. Subsequently, ALK fusion proteins were broken down into monomers with a washout of phosphate buffered salts (PBS) that removed B/B. The number of viable cells stained with trypan blue was measured daily using a Bürker-Turk calculator and the number of cells at each time point was averaged.

### Animal Experiments

Ba/F3 cells stably expressing DmrB-ALK_wt or DmrB-ALK_mF1174L, or Ba/F3 cells stably expressing EML4-ALK/EML4-ALK or EML4-ALK/EML4cc were subcutaneously injected into 8-w-old female BALB/C nu/nu mice (CAnN.Cg-*Foxn1*^*nu*^/CrlCrlj, Charles River Laboratories, Wilmington, MA, USA). For DmrB-ALK_wt or DmrB-ALK_mF1174L xenograft, subsequently, 0.5 mg/kg B/B solubilized in nano-pure water with 4% ethanol, 10% polyethylene glycol 400 (PEG-400), and 1.75% polyoxyethylene (20) sorbitan monolaurate (Tween-20), was injected twice a week into the intraperitoneal cavities of the mice. The control group received a mock solution (4% ethanol, 10% PEG-400, and 1.75% Tween-20 solubilized in nano-pure water). Tumor volumes were calculated twice a week with a modified ellipsoid formula: 1/2 × (width^2^ × length) for 5 w. In two of the six mice injected with Ba/F3 DmrB-ALK_wt, administration of B/B was discontinued after 3.5 w and changes in tumor volume were observed. The animal study protocol was approved by the Asahikawa Medical University Research Ethics Committee (#18133).

### Protein Analysis

DmrB-ALK_wt fusion proteins were extracted using an HA-tagged Protein Magnetic Purification Kit (#3342, Medical & Biological Laboratories CO. LTD. MBL. Nagoya, Japan) under non-denaturing conditions and electrophoretically separated. Protein electrophoresis was conducted by Blue-Native PAGE using Native PAGE 4-16% Bis-Tris Gel (BN1002, Invitrogen, Carlsbad, CA, USA), NativePAGE^TM^ Sample Prep Kit (BN2008, Invitrogen), NativeMark™ Unstained Protein Standard (LC0725, Invitrogen), Native PAGE^TM^ running buffer and cathode additives (BN2001, BN2002, Invitrogen), according the manufacturer's instructions. After electrophoresis, the gel was gently shaken for 30 min in SDS buffer (pH 7.7) and incubated with HA monoclonal antibodies (1:1000, #M180-3, MBL), followed by anti-mouse IgG, HRP-linked antibodies (1:5000, #7076, Cell signaling Technology). SDS-PAGE was conducted using MOPS SDS running buffer (NP0001, Invitrogen) and 4-12% Bis-Tris Gels (NP0001, NP0322, Invitrogen). Proteins were blotted using iBind^TM^ Western Systems (Thermo Fisher SCIENTIFIC) and detected by chemiluminescence (#34096, Thermo Fisher SCIENTIFIC), (#NEL113001EA, PerkinElmer, Inc., Waltham, MA, USA) (ImageQuant LAS500, GE Healthcare UK Ltd. Amersham Place, Little Chalfont, Buckinghamshire HP7 9NA, England). Cell lysis for Native PAGE and SDS-PAGE was performed using NP40 with protease inhibitor cocktails (#11836170001, Sigma-Aldrich Co. LLC, St. Louis, MO, USA).

### Protein–Protein Interactions

Fluorescent observation and flow cytometric analysis of the effect of the transfected EML4cc domain construct on endogenous EML4cc expression was performed using Fluoppi systems (#AM-8011M, MBL) according to the manufacturer's instruction. Two vectors, phAG, which has a fluorescence polarization-based tag (Azami-GFP tag) with tetramer-forming abilities, and pAsh, which has an Assembly Helper Tag (Ash-Tag) with multimerization capability from tetramer to octamer, were components of the Fluoppi systems. Fluoppi detects protein-protein interactions (PPI) as the Azami-GFP and Ash-tags assemble together to make large fluorescent foci (hereafter called Azami-GFP ^hyper^) when the tagged proteins bind to each other.

The phAG and pAsh vectors were linearized with EcoRI or HindIII (#1060A, TAKARA bio) in the multiple cloning site to create EML4-ALK constructs. The hAG (homo tetramer Azami-GFP)-tagged EML4-ALK had an Azami-GFP protein fused to the N-terminus of the EML4-ALK variant 1 (EA). The Ash (homo-oligomerized protein assembly helper)-tagged EML4-ALK had the Ash protein (oligomerization protein) fused to N-terminus of EA. The Ash-tagged EML4cc had the Ash protein fused to the C-terminus of EML4cc (cc; N′-AASTSDVQDRLSALESRVQQQEDEITVLKAALADVLRRLAISEDHVASVKKS-C′). To create Ba/F3 EA/EA cell lines, hAG-tagged EA and Ash-tagged EA constructs were co-transfected into Ba/F3 cells at a ratio of 1:1. Likewise, the Ba/F3 EA/cc cell lines were created by co-transfecting hAG-tagged EA and Ash-tagged cc into Ba/F3 cells at a vector ratio of 1:1. After electroporation, Ba/F3 cells were immediately transferred to medium containing IL-3 and incubated for 4 h. IL-3 was removed after this incubation period and cells were cultured with 1000 μg/ml geneticin (G-418 #04727878001, Roche Life Science, Basel, Switzerland) to select for positively transfected cells. After 48 h, cells were fixed in 4% paraformaldehyde, washed three times in ice cold 1 × PBS, and stored in 70% ethanol at −20°C. For flow cytometric analyses, cell nuclei were stained with propidium iodide (PI) to aid in Ba/F3 cell sorting.

### Coiled-Coil Peptides

To validate the EML4cc peptide activities, a chemically synthetic peptide was designed based on the amino acid sequence of the 3.3 kDa H-α helix structure of EML4 spanning amino acid 17T to 42K (TSDVQDRLSALESRVQQQEDEITVLK) obtained from the Protein Data Bank (https://www.rcsb.org/pdb/protein/Q9HC35, UniProtKB identifiers: Q9HC35); 1 μg/ml of EML4cc peptide was 324 nM. In this peptide, hydrophobic strands near each other are sandwiched between the hydrophilic amino acids; the burial of hydrophobic surfaces provides the thermodynamic driving force for oligomerization. Packing in the coiled-coil interface is exceptionally tight, with almost complete van der Waals contact between the side-chains of the R23 and R30 residues (Supplementary Figure 1). Lyophilized synthetic peptides (Peptide Institute, INC, Japan) were dissolved in dimethyl sulfoxide (DMSO) in 1 × PBS to achieve a concentration of 25 or 50 μg/μl. The final DMSO concentration was less than 0.1% in all experiments presented in the Results section. For visualization of intracellular trafficking of the peptide, we also made red fluorescent cc peptide (TAMRA-cc). H3122 and A549 cells were treated with the fluorescent peptides, fixed in 4% paraformaldehyde, and stained with DAPI.

To improve the cellular uptake of the peptide, we used the cell penetrating peptide (CPP) Xfect (#631324, TAKARA bio, Shiga, Japan) to use concurrently with cc peptide according to the manufacturer's instructions. In all the experiments aimed at assessing the cc peptide efficacy, the volume of Xfect used was adjusted to obtain a final concentration of cc peptide of 2 μg/ml.

### Statistical Analysis

In all cell growth assays, the mean and standard deviation were calculated from 6 wells of sample and 12 wells of control. The statistical analysis was carried out with two-sample unpaired Student *t*-test performed using R (version 3.4.1; The R Foundation, Vienna, Austria) and 95% CI of the mean difference was plotted. Comparison of survival curves was performed based on Kaplan–Meier survival analysis and log-rank test using the GraphPad Prism (version 4.60, GraphPad Software SanDiego, CA, USA).

## Results

### Effect of Conditional DmrB-ALK_wt Monomerization

**Figure 1:**
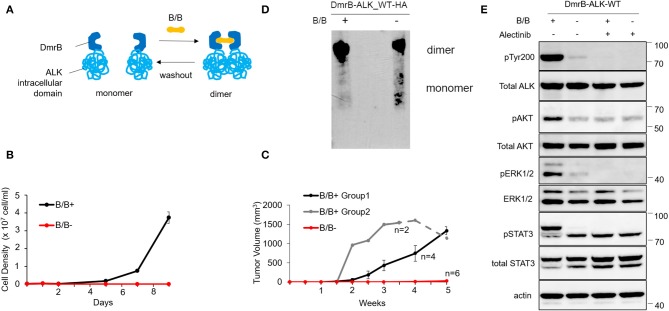
Effect of conditional monomerization of DmrB-ALK_wt. **(A)** Illustration of the inducible dimerization of wild-type ALK intracellular domain. ALK intracellular domain encoded by exons 20–29 was ligated to DmrB (DmrB-ALK_wt). Dimer/monomer state of the fusion protein was regulated by B/B homodimerizer (B/B) which acts as a ligand for DmrB. **(B)** Proliferation of Ba/F3 cells expressing DmrB-ALK_wt (Ba/F3 DmrB-ALK_wt) after B/B withdrawal or B/B continuation. Survival and proliferation of Ba/F3 DmrB-ALK_wt were dependent on B/B continuation. Error bars: SD. **(C)** Tumor volume curve of Ba/F3 DmrB-ALK_wt xenografts in nude mice. Nude mice were treated with B/B for 5 w (group 1), 3.5 w (group 2, solid line) and then withdrawn for 1.5 w (group 2, dashed line) or mock solution for 5 w. Tumor formation and growth was dependent on continuous B/B treatment. Error bars: SD. **(D)** Western blotting of the DmrB-ALK_wt protein with an anti-HA antibody. The DmrB-ALK_wt protein was immunoprecipitated with an anti-HA antibody and electrophoretically separated on a native polyacrylamide gel under non-denaturing conditions. Under the B/B treatment, monomeric DmrB-ALK_wt (predicted molecular weight of 75.5 kDa) was not observed (left lane). After withdrawal of B/B, monomeric DmrB-ALK_wt proteins were detected (right lane). **(E)** Western blotting of Ba/F3 DmrB-ALK_wt with B/B treatment (B/B+) or B/B withdrawal (B/B–). Phosphorylation of ALK, ERK1/2, AKT, and Stat3 proteins were significantly attenuated in the B/B withdrawal condition. Total and phosphorylated proteins were probed on the same blot sequentially.

Our strategy to engineer an inducible homodimer system was based on the ability of the cell-permeable rapamycin analog B/B homodimerizer to induce homodimerization of the DmrB domain [F36V variant of FKBP (26)] using the iDimerization system^TM^. We created a construct in which the DmrB domain was fused directly to the N-terminus of the *ALK* intracellular domain (exon 20–29) and transfected Ba/F3 cells, whose growth are dependent on interleukin-3 (IL-3). The dimerization of the DmrB-ALK_wt was stably expressed under the condition of B/B treatment (Figure 1A). However, Ba/F3 cells expressing DmrB-ALK_wt (hereafter called Ba/F3 DmrB-ALK_wt) with the dimerization inducer B/B proliferated without IL-3, while Ba/F3 DmrB-ALK_wt without B/B could not survive without IL-3. These results indicated that survival and growth of Ba/F3 DmrB-ALK_wt were dependent on B/B-induced dimerization (Figure 1B).

To study this interaction *in vivo*, mouse xenografts of Ba/F3 DmrB-ALK_wt were generated. Nude mice were treated with B/B for 5 w (group 1, black) or 3.5 w (group 2, gray, solid line) and then withdrawn for 1.5 w (group 2, gray, dashed line) or treated with a mock solution for 5 w (red). In the mock-treated group, tumor xenografts did not form. In group 1, tumor xenografts formed at 2 w and enlarged to an average of 1334 mm^3^ at 5 w. In group 2, tumor volume reduced after B/B withdrawal (Figure 1C). These data suggest that the growth of Ba/F3 DmrB-ALK_wt xenografts was dependent on B/B.

Next, to confirm the monomer/oligomer state of DmrB-ALK_wt, we extracted DmrB-ALK_wt protein with or without B/B from Ba/F3 cells. The DmrB-ALK_wt protein was immunoprecipitated with anti-HA antibodies and subjected to non-denaturing polyacrylamide gel electrophoresis and blotted with an HA-specific antibody. Under the B/B treatment condition, monomeric DmrB-ALK_wt (predicted molecular mass = 75.5 kDa) was not observed (Figure 1D, left lane). In contrast, after withdrawal of B/B, monomeric DmrB-ALK_wt proteins were detected (Figure 1D, right lane). To assess the status of phosphorylation of ALK and its downstream signals, Ba/F3 DmrB-ALK_wt cultured under short-term non-B/B condition, were treated with B/B and Alectinib for 2 h. The extracted proteins were electrophoresed and blotted. The phosphorylation of ALK, Erk 1/2, AKT, and Stat3 was attenuated in the non-B/B condition (Figure 1E). These data suggest that downstream ALK signaling was suppressed by monomerization of the ALK fusion protein.

### Effect of Conditional DmrB-ALK_mF1174L Monomerization

**Figure 2:**
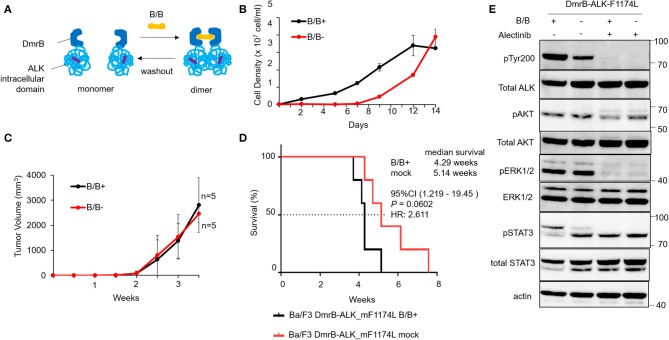
Effect of conditional DmrB-ALK_mF1174L monomerization. **(A)** Illustration of inducible dimerization of the ALK mutant F1174L intracellular domain. ALK intracellular domain with mutation from phenylalanine to leucine at amino acids 1174 of ALK (F1174L; highlighted in violet) was ligated to DmrB (DmrB-ALK_mF1174L). The dimer/monomer state of the fusion protein was regulated by B/B homodimerizer (B/B) which acts as a ligand for DmrB. **(B)** Proliferation of Ba/F3 cells expressing DmrB-ALK_mF1174L (Ba/F3 DmrB-ALK_mF1174L) after B/B withdrawal or B/B continuation. Ba/F3 DmrB-ALK_mF1174L could survive and proliferate after the withdrawal of B/B. Error bars: SD. **(C)** Tumor volume curve of Ba/F3 DmrB-ALK_mF1174L xenografts in nude mice. Nude mice were treated with B/B or mock solution for 3.5 w. Tumor xenografts were formed in both groups. Error bars: SD. **(D)** Kaplan Meier curve of Ba/F3 DmrB-ALK_mF1174L mice xenografts. Median overall survival of mice, 4 w for B/B-treated group and 5 w for mock-treated group, and was not significantly different between the groups. **(E)** Western blot of Ba/F3 DmrB-ALK_mF1174L with B/B treatment (B/B+) or B/B withdrawal (B/B–). Phosphorylation of ALK, ERK1/2, AKT, and Stat3 proteins was maintained in the B/B withdrawal condition. Total and phosphorylated proteins were probed on the same blot sequentially.

To confirm the monomeric activation of ALK point mutations that are reported as an oncogene in neuroblastoma patients (7–9), we generated an inducible dimerization construct of DmrB-ALK_mF1174L; a phenylalanine to leucine mutation at amino acid 1174 (DmrB-ALK_mF1174L; Figure 2A, highlighted in violet). In the cell growth assay, Ba/F3 DmrB-ALK_mF1174L with B/B treatment or withdrawal or maintenance were compared. Ba/F3 DmrB-ALK_mF1174L could proliferate without B/B-induced dimerization, suggesting that survival and growth of Ba/F3 DmrB-ALK_mF1174L were independent of B/B treatment *in vitro* (Figure 2B).

To study this regulatory function of protein dimerization *in vivo*, mice xenografts of Ba/F3 DmrB-ALK_mF1174L were generated. Tumor volume curves of nude mice Ba/F3 DmrB-ALK_mF1174L xenografts are shown in Figure 2C. Nude mice were treated with B/B (black), or mock solution (red) for 3.5 w. The Kaplan Meier curve of Ba/F3 DmrB-ALK_mF1174L mice xenografts indicated a median survival of 4.29 w in the B/B-treated group and 5.14 w in the mock-treated group, which was not a significant difference between these treatments (Figure 2D). The phosphorylation of ALK, Erk 1/2, AKT, and Stat3 was maintained in the B/B withdrawal condition (Figure 2E). Collectively, these data suggest that the survival and growth of the Ba/F3 DmrB-ALK_mF1174L cells were independent of B/B, indicating that the monomeric ALK-F1174L fusion proteins were oncogenic.

### Growth Inhibition of Endogenous EML4-Coiled-Coil Domain in Ba/F3 Cells Expressing EML4-ALK

**Figure 3:**
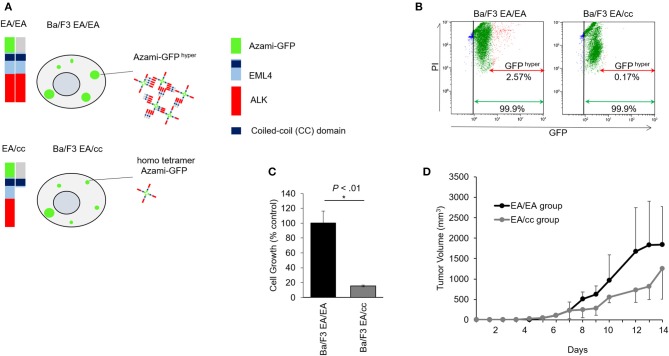
Growth inhibition of endogenous EML4-coiled-coil domain in Ba/F3 cells expressing EML4-ALK. **(A)** Schematic of EML4-ALK and EML4-coiled-coil (cc) domain interaction analysis using Fluoppi^TM^ technology. hAG-EML4-ALK, hAG (homo tetramer Azami-GFP)-tagged EML4-ALK; Ash-EML4-ALK, Ash (homo-oligomerized protein assembly helper)-tagged EML4-ALK; Ash-EML4-cc, Ash-tagged EML4-cc domain. hAG-tagged EML4-ALK (EA) and Ash-tagged EA, or hAG-tagged EA and Ash-tagged cc were co-transfected into Ba/F3 cells (Ba/F3 EA/EA and Ba/F3 EA/cc cells, respectively). Protein-protein interaction between EA-EA or EA-cc can be quantified through fluorescent intensity. In the Ba/F3 EA/EA, tagged EA proteins assemble to make oligomers, and binding of the Ash- and Azami-GFP tags makes large fluorescent foci (Azami-GFP ^hyper^). In the Ba/F3 EA/cc, Azami-GFP tagged EA proteins alone emit fluorescence (homo tetramer Azami-GFP), but only a few large fluorescent foci are formed due to Ash-hAG tag binding (Azami-GFP ^hyper^). **(B)** Flow cytometric analyses of Ba/F3 EA/EA and Ba/F3 EA/cc. In Ba/F3 EA/EA (left), all cells expressed homo tetramer Azami-GFP (green arrow) and 2.57% of cells expressed Azami-GFP ^hyper^ (red arrow). In Ba/F3 EA/cc (right), all cells expressed homo tetramer Azami-GFP (green arrow), but only 0.17% of cells expressed Azami-GFP^hyper^ (red arrow), indicating hyper fluorescent foci formation derived from Azami-GFP-EA and Ash-cc interactions was a rare fraction. X axis; GFP fluorescent intensity. Y axis; PI. **(C)** Cell growth assay of Ba/F3 EA/EA and Ba/F3 EA/cc. Ba/F3 EA/EA cells that contained oligomerized EA protein served as control. Cell growth of Ba/F3 EA/cc was significantly slower compared with Ba/F3 EA/EA. Error bars: SD. **(D)** Tumor growth assay in mice models. The animals of both Ba/F3 EA/EA and Ba/F3 EA/cc groups developed tumors. The averaged tumor growth delay of group Ba/F3 EA/cc was higher than that of group EA/EA.

To study the inhibitory effect of the endogenous EML4-coiled-coil (cc) domain in Ba/F3 expressing EML4-ALK, we created an *in vitro* fluorescent model for protein-protein interaction using Fluoppi^TM^ technology. Ba/F3 EA/EA cells expressing the hAG-tagged EA and Ash-tagged EA constructs were co-transfected in Ba/F3 cells, while Ba/F3 EA/cc cells expressing hAG-tagged EA and Ash-tagged cc constructs were co-transfected in Ba/F3 cells. Fluoppi^TM^ assays revealed a physical interaction between EML4-ALK and EML4-ALK, or EML4-ALK and EML4cc (Figure 3A).

Flow cytometric analyses of Ba/F3 EA/EA and Ba/F3 EA/cc cells are shown in Figure 3B and Supplementary Figure 2. Ba/F3 cells expressing Azami-GFP and treated with PI were sorted and compared with untreated Ba/F3 to generate nuclei stained (Gate B in Supplementary Figure 2) and GFP positive (Gate C in Supplementary Figure 2) populations. When Ba/F3 EA/EA cells were sorted, an intense GFP signal was observed and determined as Azami-GFP ^hyper^ (Gate D, Supplementary Figure 2). In Ba/F3 EA/EA cells (Figure 3B, left panel), 99.9% of cells expressed Azami-GFP and 2.57% of cells showed Azami-GFP ^hyper^, wherein the interaction between the Azami-GFP-EA proteins and Ash-EA proteins produced hyper-fluorescent foci. In Ba/F3 EA/cc cells (right panel), 99.9% of the cells expressed Azami-GFP, but only 0.17% of the cells showed Azami-GFP ^hyper^. This indicates that homo tetramer Azami-GFP-EA protein was a major population, and Azami-GFP ^hyper^, which reflected the interaction between Azami-GFP-EA protein and Ash-EML4cc protein, was only a rare fraction of interactions after 48 h of gene introduction. In the MTS cell growth assay, Ba/F3 EA/cc cell growth was significantly lower than that of Ba/F3 EA/EA, indicating that EA oligomerization by endogenous cc expression could inhibit tumor growth (Figure 3C). In the xenograft model, inoculated Ba/F3 EA/EA and Ba/F3 EA/cc developed tumors; tumor growth delay was more pronounced in the Ba/F3 EA/cc group than in the EA/EA group (Figure 3D).

### EML4-cc Peptides Inhibit Growth of H3122 and Ba/F3 Cells Expressing EML4-ALK

**Figure 4:**
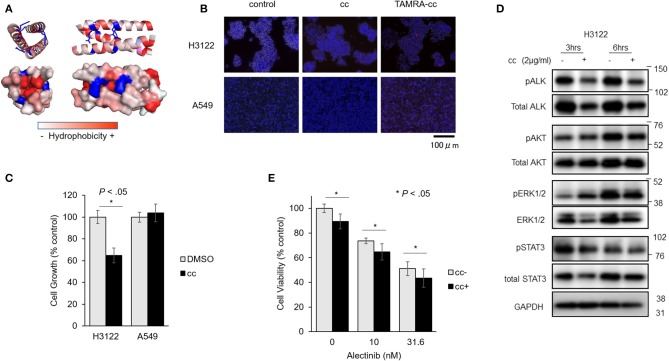
EML4-cc peptides inhibit growth of H3122 and Ba/F3 cells expressing EML4-ALK. **(A)** Peptide sequence and 3D structure of EML4cc constructs. The EML4cc peptide consists of the original EML4-coiled-coil domain spanning amino acids T17 to K42 and were chemically synthetized. The 3D structure of EML4cc was generated by PyMOL2.0.6. **(B)** H3122 and A549 cells were treated with PBS, EML4cc peptide, or EML4cc conjugated with TAMRA (red fluorescence). After 6 h of treatment with PBS or 1 μg/ml of EML4cc peptides, the cells were observed under a fluorescence microscope. Red fluorescence of TAMRA was detected in the cytoplasm of both H3122 and A549 cells. **(C)** Cell growth of H3122 and A549 cell lines. EML4cc peptides inhibited the growth of H3122 cells, but not that of A549 cells. Error bars: SD. **(D)** Western blotting of H3122 extracts treated with cc peptides. The phosphorylation of ALK was decreased at 3 h after peptide treatment; the same trend was visible at 6 h. The phosphorylation of ERK and AKT was decreased at 6 h after cc peptide treatment. **(E)** The efficacy of the combination of Alectinib and cc peptides. After 24 h from concurrent administration of Alectinib and cc peptide treated with Xfect, the viability of H3122 cells treated with Alectinib and cc peptide was significantly decreased in comparison with that of cells treated with Alectinib alone. Error bars: SD.

We next tested whether EML4cc peptides can limit growth of tumor cells with *EML4-ALK* rearrangements. Schematics of peptide sequences and 3D structure of EML4cc are presented in Figure 4A. The EML4cc peptide, consisting of the EML4-coiled-coil domain from residues T17 to K42, was chemically synthetized. A 3D structure of EML4cc was generated by PyMOL2.0.6. The EML4cc structure was obtained from Protein Data Bank (PDB_4CGC). The peptide was colored from red to white to indicate amino acid hydrophobicity scales. The V20, L24, L27, and V31 positions were hydrophobic, thus stabilizing helix oligomerization through hydrophobic and van der Waals interaction. Side chain residues R23 and R30 are charged in order to form interhelical electrostatic interactions.

To study the cellular uptake of cc peptide in cancer cell lines, H3122 and A549 cells were treated with cc peptides. To monitor the uptake, the cc peptide was conjugated with TAMRA (red fluorescence). After 6 h of treatment with 1 μg/ml of cc peptide, the fluorescence signal of TAMARA-cc was detected in the cytoplasm of both H3122 and A549 cells (Figure 4B). Especially in H3122 cells, red clusters were observed within the cytoplasm.

To confirm cc peptide-induced cytotoxicity, H3122 and A549 cells were treated with the cc peptides with Xfect for 24 h. In the MTS cell growth assay, the cc peptide administered at 2 μg/ml significantly reduced the growth of H3122 cells to 63% (with respect to Xfect + DMSO control) and did not induce cell growth inhibition in A549 cells (Figure 4C).

To evaluate the status of phosphorylation of ALK and its downstream signals, H3122 cells were treated with cc peptide with Xfect for 3 and 6 h, and whole proteins were extracted and blotted. The phosphorylation of ALK was decreased at 3 and 6 h post-treatment, and the phosphorylation of ERK and AKT was decreased 6 h after cc peptide treatment (Figure 4D). These data suggest that cc peptide could induce reduction of ALK and activation of its downstream survival signals.

We examined the cc-peptide and Alectinib combination therapy in H3122 cells. At 24 h after concurrent administration of Alectinib and cc peptide in combination with Xfect, the viability of H3122 cells treated with Alectinib and cc peptide was significantly decreased in comparison with that of cells treated with Alectinib alone (Figure 4E).

## Discussion

Previous reports suggest that the oncogenic EML4-ALK fusion protein requires ALK homodimerization via trimeric coiled-coil (cc) domains present in the amino-terminal of EML4 for constitutive ALK activation (4, 5, 10–12). In our DmrB-ALK_wt model, which allows dimerization to be disrupted, phosphorylation of ALK and its downstream signals significantly decreased so that tumor growth was completely suppressed both *in vitro* and *in vivo*. In the first report of EML4-ALK in NSCLC, Soda et al. also reported that the deletion of the EML4 basic region that consists of the cc domain does not lead to tumor formation in a mouse xenograft model (4). In neuroblastoma, ALK amplification and gain-of-function mutations were found within the ALK tyrosine kinase domain including F1174L or R1275Q (27), the resulting protein could contribute to ALK autophosphorylation and activate downstream signaling in a monomer state (9). Previously, we also reported the ALK-F1174L mutation in acquired resistant patients with inflammatory myofibrotic tumors after ALK-TKI treatment (6). In the DmrB-ALK_mF1174L model, we showed that tumors rapidly grew regardless of its monomeric or oligomeric state *in vitro* and *in vivo*. These data suggest that the phosphorylation of ALK and activation of its downstream signals were maintained even after the ALK intracellular domain was monomerized. From these data, we sought to determine whether therapeutic monomerization of ALK fusion proteins is a valid anti-cancer strategy for tumors without ALK gain-of-function mutations.

The methodology for therapeutic monomerization of intracellular ALK fusion proteins using exogenous substances was challenging. Firstly, we attempted to create small molecules (molecular weight < 500) to directly fragment the trimeric EML4cc for EML4-ALK dissociation, because small molecules benefit from their ability to penetrate cell membranes and easily reach intracellular targets (28). For example, some ALK-TKIs used for the treatment of ALK positive NSCLC are small molecules and show clinically good results (29). However, in our simulation, it was impossible to design a small molecule that can directly tear-off the coiled-coil assembly. As an alternative approach, we focused on peptide-based therapeutics, because they are potent and selective against biological targets that are otherwise difficult to manipulate with small molecules. Therefore, we investigated the use of mimicking peptide therapy targeting the EML4cc domain as a monomerizing agent in reference to the study of leukemia. Previous reports show that in Bcr-Abl (Philadelphia) t(9;22) chromosome translocated leukemia, overexpression of the Bcr oligomerization domain or the mimicking peptide therapy aimed at oligomer dissociation of the Bcr-Abl protein demonstrated distinct anti-cancer activities in leukemia cell lines and some tumors resistant against Abl kinase inhibitors (18, 21–25). In our present study, endogenous EML4cc expression reduced EML4-ALK self-assembly and suppressed tumor cell growth *in vitro* and *in vivo*, suggesting that overexpression of the EML4-cc domain induced oligomer dissociation of the EML4-ALK protein. We also demonstrated that exogenous administration of synthetic EML4cc peptides suppressed ALK phosphorylation and tumor cell growth in EML4-ALK positive cells. When treated with Alectinib, the cc peptide administration showed stronger inhibition than ALK-TKI single treatment. These observations convinced us that monomerization of ALK fusion proteins as a therapeutic strategy in ALK-Rearranged Non-small Cell Lung Cancers.

Peptide dynamics and *in vitro* and *in vivo* stability, including self-aggregation of the cc peptide itself, association with native EML4, and cell membrane permeability are potential challenges to address before peptide therapies can be considered as treatment options. In order to improve cell membrane permeability or to confer stability, modification of the peptide with a cell penetrating peptide (CPP) or staple peptide should be investigated. CPP can introduce drugs efficiently and selectively into tumor tissues, thereby maximizing their therapeutic effects; for example, polyhistidine (H16) administered to mice human fibrosarcoma xenografts accumulated in tumor tissue *in vivo* (30). Stapled peptide refers to a peptide whose side chain has been cross-linked (stapled) in order to stabilize the secondary structure of the peptide; this improves the binding constant and membrane permeability. The staple peptide is under development in the treatment of cancers such as chronic myeloid leukemia, but not yet in lung cancers (31–33). These types of modifications might enhance the anti-tumor effects of our EML4cc peptide against EML4-ALK fusion positive cancers, and are being considered for future studies.

In conclusion, this is the first report to demonstrate the therapeutic potential of inducing monomerization of the ALK fusion proteins through use of a competing the mimetic peptide of EML4cc. Further studies are warranted to explore the use of specific cc peptide as a therapeutic option for other lung cancers harboring driver fusion genes containing an oligomerization domain.

## Data Availability Statement

All datasets generated for this study are included in the article/Supplementary Material.

## Ethics Statement

The animal study was reviewed and approved by the Asahikawa Medical University Research Ethics Committee.

## Author Contributions

NH and TS conceived and designed the study, provided data analysis, and interpretation. NH, TS, and SC provided administrative support and provision of Hirai et al. ALK Monomerization as Cancer Therapy study materials. NH collected and assembled all of the data. All authors contributed to the writing of the manuscript and provided final approval of it.

### Conflict of Interest

The authors declare that the research was conducted in the absence of any commercial or financial relationships that could be construed as a potential conflict of interest.
