# Supplementary material for: Monomerization of ALK Fusion Proteins as a Therapeutic Strategy in ALK-Rearranged Non-small Cell Lung Cancers
Source: Front Oncol. 2020 Apr 2;10:419. doi: 10.3389/fonc.2020.00419 (PMC7142238; doi:10.3389/fonc.2020.00419)
Supplement: Supplementary file 1 [file Data_Sheet_1.docx]

Supplementary Material

## Supplementary Figures


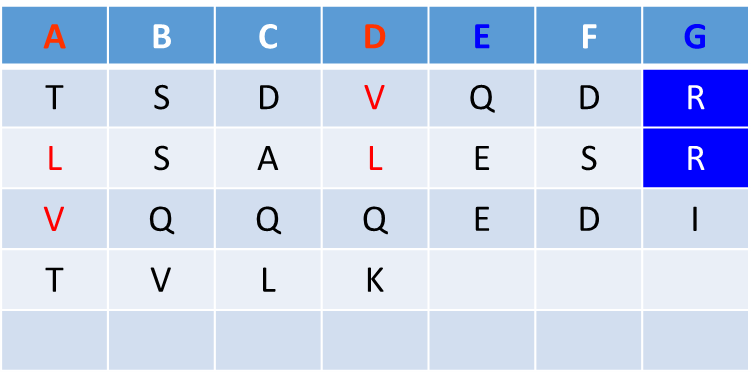


**Supplementary Figure 1.** Peptide sequence of EML4cc constructs. Positions in the heptad repeat are labeled ABCDEFG. Columns A and D represent those usually containing hydrophobic amino acids. Columns E and G represent those containing charged amino acid residues.


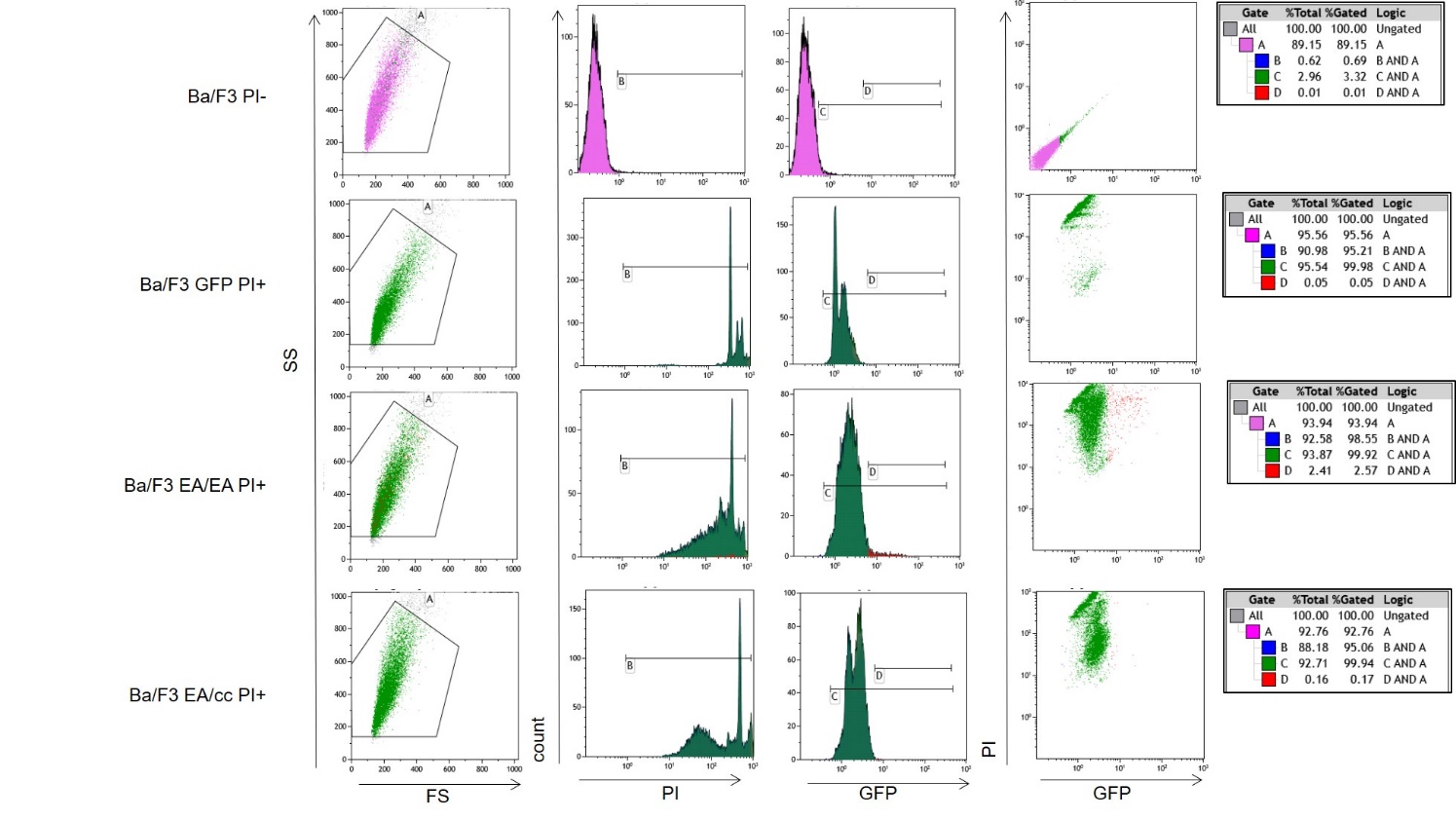


**Supplementary Figure 2.** Flow cytometric analyses of Ba/F3 cells without PI staining, and Ba/F3 GFP, Ba/F3 EA/EA, and Ba/F3 EA/cc cells with PI staining. Ba/F3 cells expressing Azami-GFP and stained with PI were sorted and compared with untreated Ba/F3 to generate nuclei stained population (Gate B) and GFP positive population (Gate C). When Ba/F3 EA/EA cells were sorted, an intense signal of GFP was observed and determined as Azami-GFP ^hyper^ (Gate D). The % total or % Gate A fraction (% Gated) of cells of each cell line is shown in the right panel.
